# Exposure to Pb impairs breeding success and is associated with longer lifespan in urban European blackbirds

**DOI:** 10.1038/s41598-018-36463-4

**Published:** 2019-01-24

**Authors:** Clémentine Fritsch, Łukasz Jankowiak, Dariusz Wysocki

**Affiliations:** 1 Chrono-environnement, UMR 6249 CNRS / University Bourgogne Franche-Comté Usc INRA, 16 route de Gray, F-25030 Besançon Cedex, France; 2 Department of Vertebrate Zoology and Anthropology, University of Szczecin, Wąska 13, 71-412 Szczecin, Poland

## Abstract

Although several factors have been highlighted to explain the influence of urbanization on bird fitness and survival, the role of persistent toxicants such as lead (Pb), which is typically present in urban areas worldwide, has seldom been studied despite the ecological importance of such a widespread stressor. Studying free-living European blackbirds (*Turdus merula*) in city parks, we tested the hypothesis that low-dose chronic exposure to Pb could shape the life-history traits of urban birds. The feather concentrations of Pb and cadmium were typical of urban areas and low-to-moderate contamination of sites. Although the lifetime breeding success of females decreased with increasing exposure to Pb, the lifespan and survival probabilities of blackbirds increased with Pb contamination regardless of gender. Breeding effort-dependent patterns in the relationship between lifespan and Pb levels were highlighted. No significant relationships were detected between cadmium and life-history traits. The results suggest a possible trade-off between self-maintenance and reproduction, with the most affected birds redirecting allocations towards their own survival, which is consistent with the “*stress hormone hypothesis*”. These findings suggest that Pb pollution in urban environments may shape avian ecological features and be one of the drivers of wildlife responses to urbanization and that some urban areas may function as ecological traps driven by pollutants.

## Introduction

Urbanization represents a major concern related to global change given the current trends in urban population growth and urban land cover expansion and is one of the most irreversible anthropogenic impacts on the global biosphere^1–3^. Urbanization leads to modifications in animal behaviour, phenology, morphology, genetics, population dynamics, community structure and selection pressures^2,4,5^. Urban areas may attract wildlife due to relaxed pressures in comparison with rural or forest habitats such as food availability and predictability throughout the year, reduced predation rates and reduced parasite pressure, a prolonged breeding season and the lack of a need for migration^6,7^. The overall picture arising from the literature is that avian survival may increase, whereas productivity decreases in urban areas^7–9^. Several drivers of this trend have been highlighted such as human disturbance, the poor nutritional quality of resources, habitat fragmentation, predation and competition as well as light and noise pollution^8–10^. Nonetheless, knowledge about the potential effect of chronic exposure to the chemical pollutants usually present in urban environments on bird demographics and life-history traits is lacking.

Urban ecosystems worldwide are typically contaminated with lead (Pb)^11–13^. Lead may threaten human and wildlife health and constitute a new evolutionary force, thus representing a major environmental issue in urban ecosystems^14,15^. Lead contamination is still a concern despite the dramatic decrease of atmospheric emissions during recent decades^16,17^. Because Pb is persistent, the legacy of contamination remains in urban soils, and although the current atmospheric emissions are significantly decreased, they are far from null^13,16,18–21^. Urban birds have been shown to be exposed to Pb worldwide, although the levels rarely reach the acute exposure found at heavily polluted sites (see, for example, some recently published studies)^13,18,22,23^.

Lead is a non-essential trace metal and is highly toxic because it acts as a nonspecific toxin affecting normal biological functioning via oxidative stress and its ability to substitute for other essential ions^24^. Harmful effects of Pb on birds have been reported, including the impairment of breeding success and survival, and endocrine disruption^25–27^. Negative correlations between Pb and reproductive success and nestling condition and survival were found in early studies in cities before the effective decrease in Pb emissions^28–30^. Most of the studies on Pb in terrestrial avian wildlife have been conducted in severely polluted areas, whereas the current level of pollution in cities has seldom been considered, and little is known about the effects of chronic low-dose Pb exposure despite the potential ecological importance of such a widespread stressor^23,31^. Eeva *et al*. (2006) proposed a “*stress hormone hypothesis*” to explain why male pied flycatchers (*Ficedula hypoleuca*) showed a higher survival probability in a metal-polluted area, stating that metal pollution might affect stress hormone levels, triggering a redirection of investment towards their own survival instead of reproduction and/or a modification of territoriality or breeding dispersal^32^. The life-history traits of birds exposed to sub-lethal levels of Pb in cities might thus be modified, and a reallocation of resources to self-maintenance at the expense of reproduction could be expected with gender-related patterns.

Could exposure to Pb be involved in shaping the ecology of urban birds? Our purpose was to investigate whether chronic low-dose exposure to Pb in urban environments could be a driver of life-history traits in the European blackbird, *Turdus merula*. This omnivorous passerine bird is considered an urban adapter^33^, it has been reported to exhibit differences in productivity and survival between urban and non-urban areas^8,9^, and can be exposed to Pb through trophic transfer within food webs^34^. To avoid the potential influence of confounding factors that often occur when comparing urban birds to their rural counterparts, an urban blackbird population was studied and traits were related to individual Pb exposure. Exposure to Cadmium (Cd), another noxious trace metal often present with Pb in urban soils^23,34^, was also evaluated to provide further insight into the role of Pb with regard to global metal pollution. If the current levels of Pb pollution in the city are a constraint on bird life-history traits, we predict that (1) the levels of Pb in feathers will be characteristic of areas with low-level pollution and close to toxicological benchmarks, and (2) relationships will exist between exposure to Pb or Cd and life-history traits. Specifically, if Pb-induced stress triggers a trade-off between self-maintenance versus reproduction, we predict that reproductive success and parental investment will decrease with exposure to Pb, whereas survival will increase and the relationship between productivity and lifespan will be shaped according to Pb exposure levels. According to the “*stress hormone hypothesis*” proposed by Eeva *et al*. and the results from recent studies, males should be more affected than females^31,32^; however, other studies have stressed a greater sensitivity of females towards Pb-induced impacts^35–37^. Therefore, gender-related differences in responses are also expected, but their direction is unpredictable.

## Results

### Trace metals in feathers

**Figure 1:**
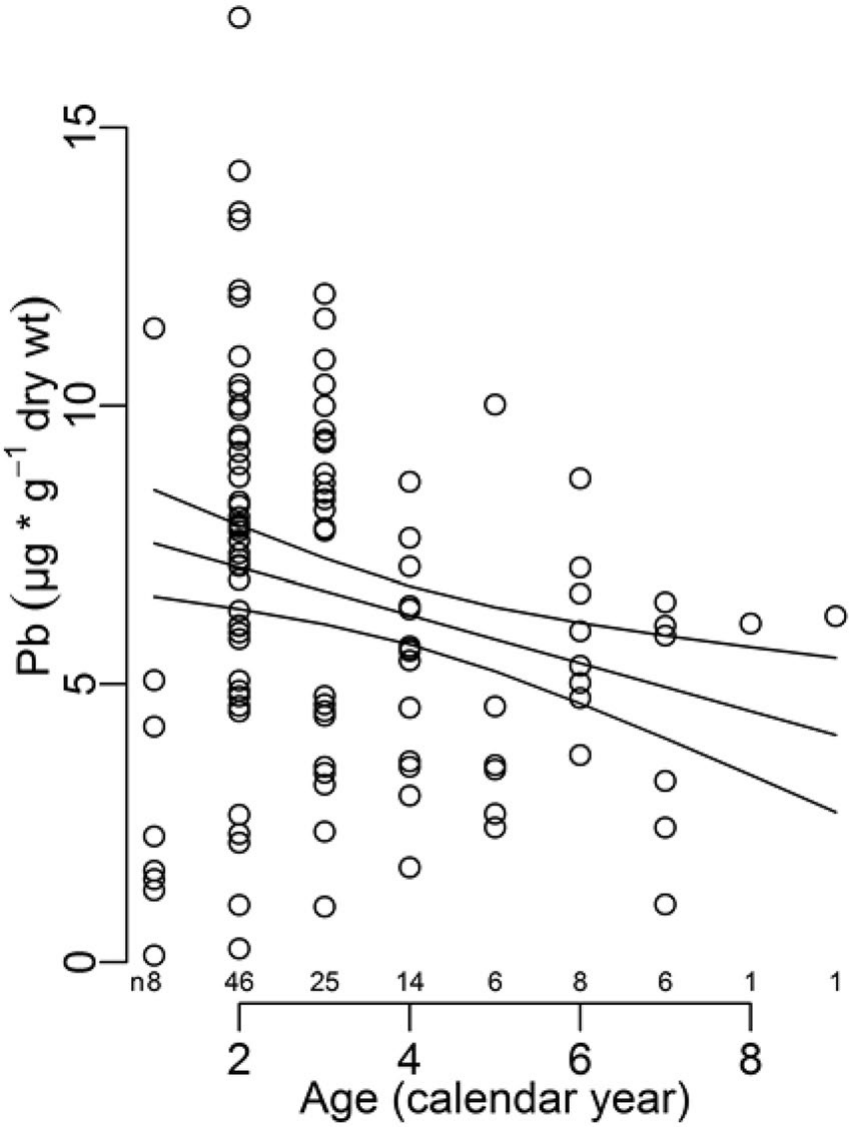
Relation between Pb exposure and age in blackbirds. Concentration of Pb in tail feathers; the enveloping lines are 95% confidence intervals, *n* = 115. Numbers above the x-axis indicate the sample size of the different age groups.

The levels of metals in feathers are provided in Table 1. A negative linear trend was found between the Pb levels in tail feathers and age (Fig. 1, *n* = 115, GLS: β_0_ = 7.956 ± 0.605; β_age_ = −0.430 ± 0.133; *t* = 3.22, *p* = 0.0017, variance explained = 3.4%), indicating that older birds exhibited lower levels of feather contamination than younger birds. We did not find any significant effect of age on Cd contamination (Supplementary Information A1: Table A3).

**Table 1:** Trace metal concentrations in feathers of blackbirds (µg.g^−1^ DW).

	Tail feathers			Wing feathers		
	All	Females	Males	All	Females	Males
Żeromski Park						
*n*	115^+^	63	51	56	25	31
Cd	0.22 ± 0.15	0.24 ± 0.18^a^	0.20 ± 0.11^a^	0.21 ± 0.13	0.18 ± 0.09^a^	0.23 ± 0.15^a^
Pb	6.7 ± 3.8*	6.8 ± 4.1^a^	6.6 ± 3.6^a^	3.8 ± 2.0*	3.2 ± 1.8^a^	4.3 ± 2.1^a^
Cemetery park						
*n*	20	9	11			
Cd	0.26 ± 0.17	0.22 ± 0.10	0.29 ± 0.21			
Pb	4.0 ± 2.4	4.1 ± 3.4	3.9 ± 1.4			

Statistics were performed on the data from Żeromski Park. For a given element, differences between feather types for all birds are indicated by an asterisk. For a given element and a given type of feather, differences between males and females are indicated with letters. Values that share a similar letter do not significantly differ (*p* > 0.05). ^+^Indicates that the individual gender of all individuals is not known.

### Breeding success and trace metals

**Figure 2:**
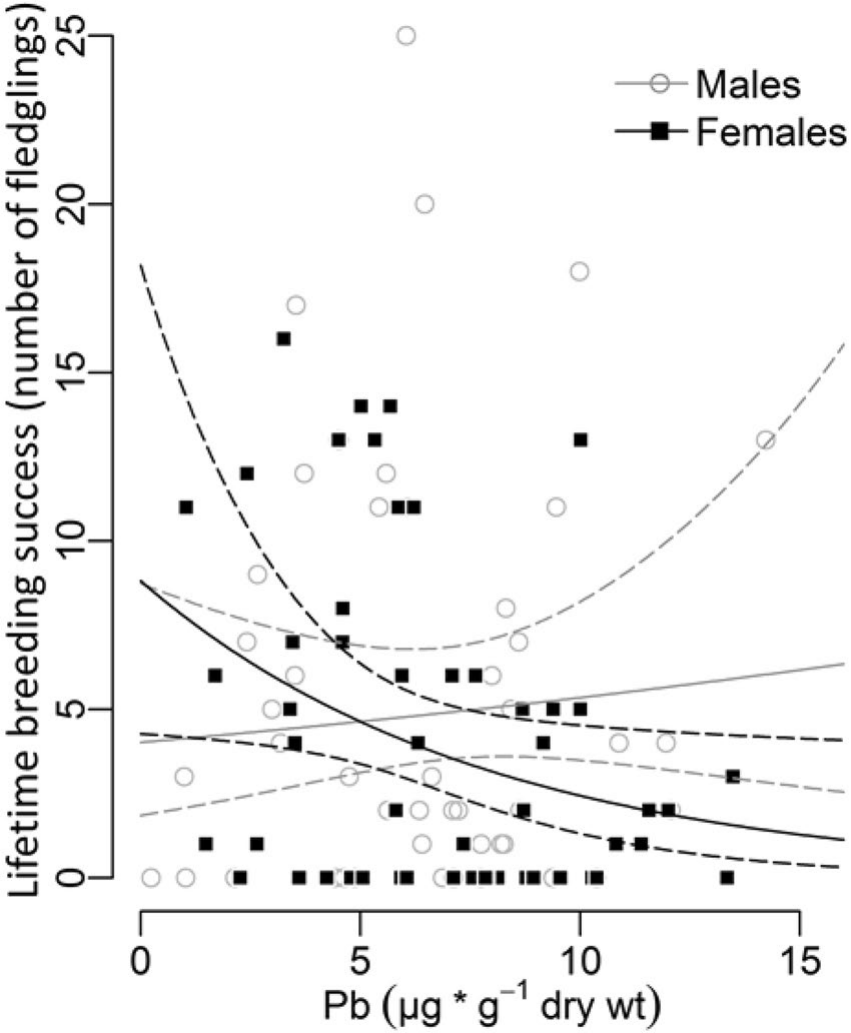
Relation between lifetime breeding success and Pb exposure in blackbirds. Lifetime breeding success corresponds to the number of fledglings. Concentrations of Pb in tail feathers; the enveloping lines are 95% confidence intervals, *n* = 108.

The lifetime breeding success of females decreased with increasing Pb levels in tail feathers, whereas no such decline was observed for males (Fig. 2; Table 2). No significant relationship with lifetime breeding success was detected for Cd (Table 2). Pb and Cd levels were not significantly related to breeding success in the year of feather sampling or brood number variables (the number of broods in the year of sampling, lifetime number of broods) (*n* = 114, *p* > 0.05, Supplementary Information A1: Tables A4–A6).

**Table 2:** Statistical outputs for lifetime breeding success and trace metal concentrations in tail feathers.

	Estimate	SE	t-value	Pr (>|t|)	% of deviance explained
(Intercept)	0.524	0.283			
sex[m]	−0.240	0.400	−0.600	0.550	0.21
Pb	−0.118	0.046	−2.529	0.013*	**3.65**
Cd	0.198	0.817	0.243	0.809	2.38
Pb:sex[m]	0.141	0.062	2.284	0.025*	**5.97**
Cd:sex[m]	−2.785	1.552	−1.795	0.076	3.57

Generalized linear models using a quasipoisson distribution and offset variable lifespan of the bird with sex differences, *n* = 108). Significant results (*p* < 0.05) are marked in bold with an asterisk*.

### Survival and trace metals

**Figure 3:**
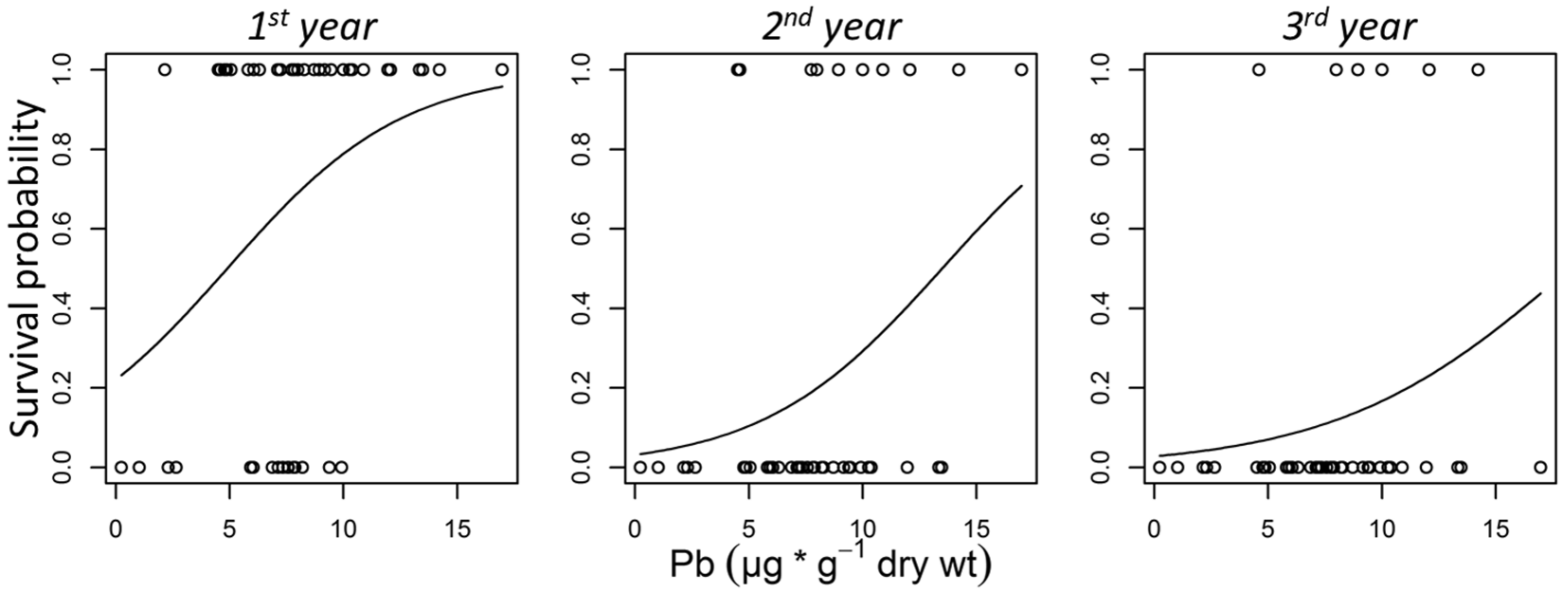
Survival of young blackbirds in relation to exposure Pb. Survival for the first, second and third year following feather sampling versus Pb concentration in the tail feathers of young blackbirds in their second year of life, *n* = 46.

The survival probability of young birds (*n* = 46) in the 1^st^ year after feather sampling significantly increased with Pb levels (*n*_survived_ = 30 [65%], β_0_ = −1.260, β_Pb_ = 0.257 ± 0.117; *z* = 2.204, *p* = 0.028) as did survival in the 2^nd^ year after feather sampling (*n*_survived_ = 10 [22%], β_0_ = −3.416, β_Pb_ = 0.253 ± 0.123; *z* = 2.055, *p* = 0.040) (Fig. 3); this relationship became non-significant in the 3^rd^ year after sampling (*n*_survived_ = 6 [13%], β_0_ = −3.551, β_Pb_ = 0.194 ± 0.134; *z* = 1.451, *p* = 0.147). Cd levels did not significantly affect the probability of survival (Supplementary Information A1: Table A7–A9).

**Figure 4:**
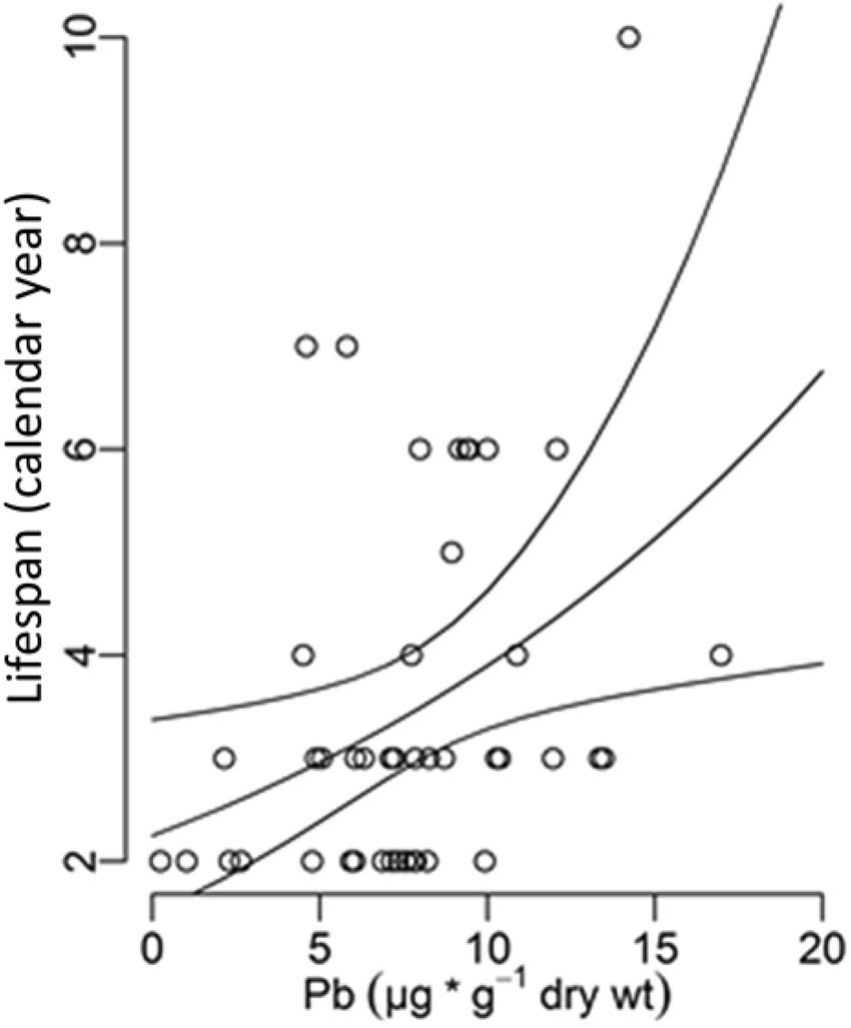
Relation between lifespan of young blackbirds and Pb exposure. Concentration of Pb in tail feathers for young blackbirds in their second year of life, *n* = 46. The enveloping lines are 95% confidence intervals.

A significant relationship was found between Pb contamination and the lifespan of birds sampled in their second calendar year of life, with the most contaminated birds of both sexes living longer (Fig. 4, *n* = 46; β_0_ = 0.810 ± 0.207, β_Pb_ = 0.055 ± 0.022; z = 2.403, p = 0.0163, variance explained = 15%). We did not detect any significant relationship between lifespan and Cd contamination (Supplementary Information A1: Table A10).

### Trade-off between breeding effort and survival

**Figure 5:**
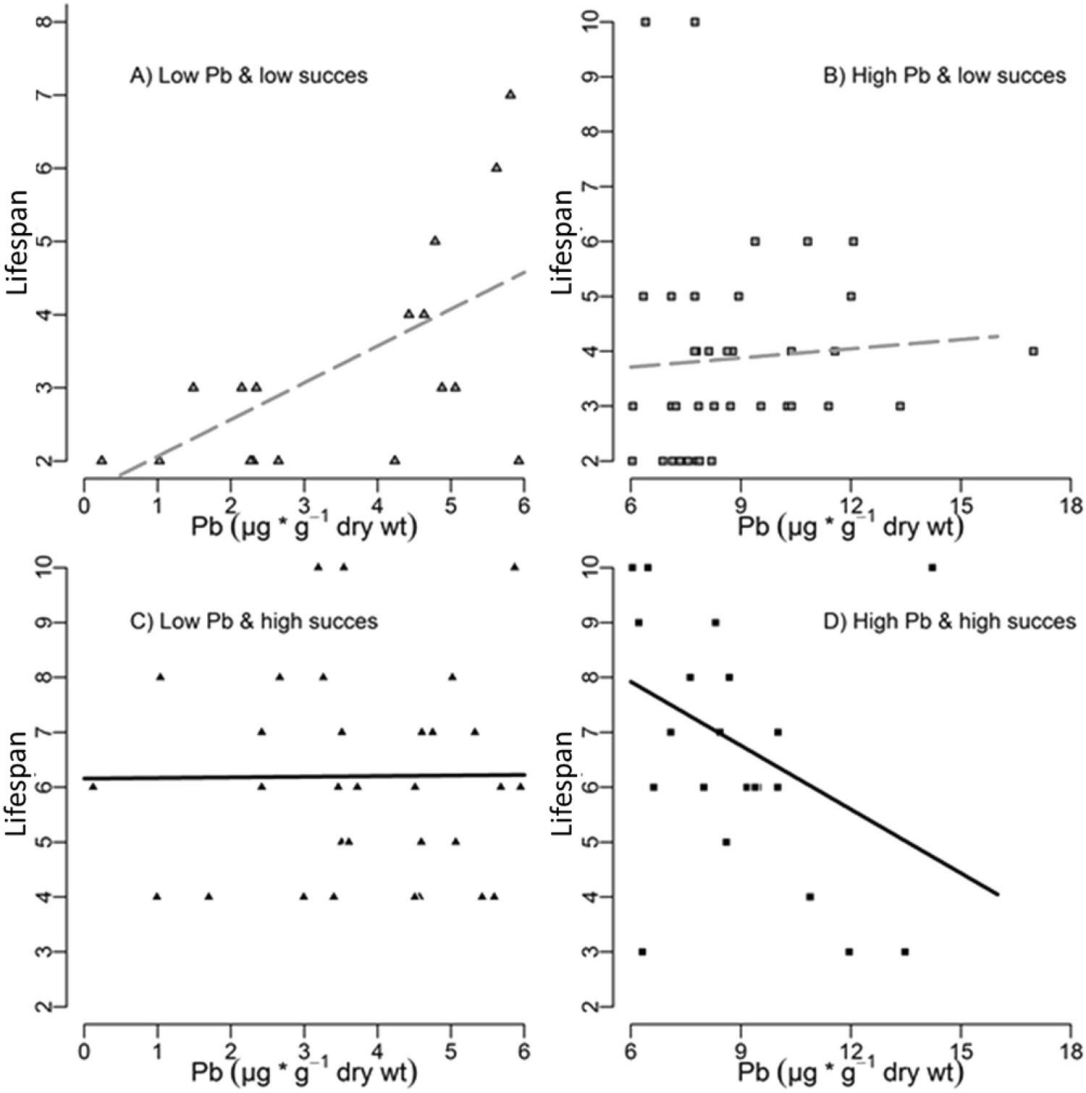
Overview of lifespan variation with Pb in feathers: Breeding effort and level of Pb exposure. Level of Pb exposure: low = [Pb]_tail feather_ < 6 ppm, high = [Pb]_tail feather_ ≥ 6 ppm. Breeding effort: low = lifetime fledgling production <3, high = lifetime fledgling production > 3.

In birds with the greatest exposure and the highest breeding success, breeding effort can be considered detrimental because their lifespan tended to decrease with increasing contamination (Fig. 5, *n* = 23, β_0_ = 2.44 ± 0.33, β_Pb_ = −0.060 ± 0.04, *p* = 0.11, variance explained = 14.1%); such a relationship was not detected for birds with lower exposure (*n* = 30, β_0_ = 1.82 ± 0.20, β_Pb_ = 0.001 ± 0.05, *p* = 0.97, variance explained <0.1%). In birds with low breeding success, lifespan increased with increasing Pb contamination, suggesting that a reduced breeding effort has a protective effect on the negative effects of Pb, but this effect was statistically significant only at low levels of contamination (*n* = 18, β_0_ = 0.61 ± 0.34, β_Pb_ = 0.16 ± 0.08, *p* = 0.049, variance explained = 37%). For highly contaminated birds with low fledgling production, we did not find any significant relation (*n* = 38, β_0_ = 1.23 ± 0.33, β_Pb_ = 0.01 ± 0.4, *p* = 0.70, variance explained = 0.5%).

### Parental investment and trace metals

Neither Pb nor Cd levels in feathers were significantly related to the start or end of the breeding season in the year of feather sampling (*n* = 89, Supplementary Information A1: Tables A11–A12). We did not detect any differences between males and females in the probability of abandonment of the last clutch (*n* = 89), but due to heavy predation, the sample size was small (14 broods abandoned by 8 males and 6 females), rendering this result inconclusive. When considering the breeding season of feather sampling as well as the year before and the year after, the difference was still not significant, although the proportion of birds abandoning fledglings was higher for males (35% males, *n* = 20 and 16.7% females, *n* = 24; Fisher exact test, *p* = 0.15). Additionally, there were no differences in fledgling abandonment between “highly” and “lightly” contaminated birds.

## Discussion

As expected, the levels of exposure of these urban blackbird populations to trace metals are typical of sites with low-to-medium pollution. Indeed, although direct comparisons of the site and feather levels should be considered with caution depending on the type of feather sampled and the washing procedure, both Cd and Pb levels are within the range of concentrations measured in the feathers of blackbirds from areas with low pollution and from current urban areas^23,34,38–40^. Far greater levels of metals (more than 50 and 5 ppm DW for Pb and Cd, respectively) have been reported in the feathers of blackbirds sampled in heavily polluted areas^34,38^. Pb concentrations depend on the type of feather analysed, with greater levels reported in the largest feathers (i.e., tail feathers), and potential variation due to the timing of moulting is consistent with the literature^41^.

The measurement of Pb concentrations in the blood is a proxy for short-term exposure to lead, whereas Pb concentration in feathers reflects subchronic exposure during the time of feather growth^42,43^. However, studies on sedentary blackbirds from reference, polluted or urban sites show strong correlations between the levels of Pb in blood and in feathers^34,38,40^, indicating that the individual level of exposure in a given year (measured in blood) is consistent with the chronic level of exposure the year before (assessed in feathers). Data from other studies thus allow the expected levels of Pb in the blood to be extrapolated from the Pb concentrations in feathers. Although such computed values cannot be considered an accurate reflection of exposure concentrations, they provide an expected range of Pb blood levels that allow further insights into the toxicological significance of blackbird exposure to Pb. Based on this extrapolation, 29–72% of the blackbirds may exceed the value of 20 μg.dL^−1^ Pb in the blood, which is a benchmark related to subclinical effects in birds^26^. Given the recently proposed benchmark of 71 µg.dL^−1^ ^44^, 2–21% of the blackbirds may suffer from deleterious effects of Pb. Based on the data from wing feathers (the same calculation was performed for wing feathers using data from Fritsch *et al*. (2012)), 81% and 22% of the blackbirds are likely to exceed the toxicological benchmarks of 20 and 71 μg.dL^−1^, respectively. Furthermore, 0–1% of the birds are likely to exceed the value of 200–300 μg.dL^−1^ Pb in the blood, which is the threshold proposed for clinical or severe clinical poisoning in birds^26^.

The levels found here are within the range of Pb concentrations in feathers associated with an increase in feather corticosterone levels in blackbirds sampled along an urbanisation gradient, where Meillère *et al*. (2016) suggested that the urbanisation constraint on birds could be mediated by trace elemental pollution. Some other effects of Pb could occur even at such low levels of exposure, including oxidative stress, a decrease in δ-aminolevulinic acid dehydratase levels in blood, a depletion of vitamins and essential elements, the disruption of cognition and behaviour, and an increase in the prevalence of pathogens^22,45–48^. Sub-lethal exposure to Pb is likely to induce endocrine disruption in birds, affecting the levels of hormones involved in the breeding effort and responses to stress such as changes in the levels of corticosterone and testosterone^23,25–27,31,49,50^, which are also thought to mediate the trade-off between survival and reproduction^51–53^. Subclinical effects of Pb can therefore be expected in this population, with a probable modulation of physiology and behaviour by stress hormones. Our findings strongly suggest that a significant portion of the blackbirds living in this city, and thus in many urban parks around the world, may be subject to the detrimental effects of Pb with subclinical responses to this stressor.

The fact that bird exposure to metals in this study is assessed on a single measurement may bear comment. Because this is not a central topic of the discussion for this study, only the main issues are summarized hereafter, but an addendum to the discussion is provided with further details (Supplementary Information A2). Given the biology of the species, the environmental trends in Pb, the data in this study, and results regarding the current level of exposure of birds to Pb in cities, the exposure to Pb pollution in this urban park can be expected to be chronic from early life onward. Furthermore, it is possible that early exposure to Pb during the first weeks or months of life could affect the neurological development and the fitness of the birds during their entire life, even if the level of exposure decreased during adulthood. The time gap between the exposure assessment and the breeding parameters cannot consequently be considered as a bias. This renders a single measure of contamination over time appropriate for exposure assessment and for investigating the relationships with life-history traits. Nevertheless, we acknowledge that the repeated assessment of Pb (and Cd or other pollutants) exposure would improve our investigations and provide a better understanding of the chronic exposure patterns in cities as well as the responses of the birds over the short-, mid-, and long-terms. This drawback should be addressed in further studies. Future studies should also conduct analyses of several feathers per bird to address sampling heterogeneity. Furthermore, to better control for external contamination, future studies should also exclude barbs from feather shafts prior to trace element analyses^43^.

The negative impacts of Pb on avian reproductive success have been well documented^26–28^. The present results are partly in accordance with previous studies, with a decrease in lifetime breeding success with increasing exposure to Pb, whereas other breeding parameters were not significantly related to metal levels. Chronic low-dose exposure to Pb in urban areas can thus negatively influence breeding success, but these deleterious impacts may be less obvious than in highly polluted areas, requiring long-term observation to detect effects against background and other urban stressor-induced variations.

Owing to the demonstrated noxious effects of Pb on avian health and survival^26,27^, the increase in survival with increased exposure to Pb can be considered unexpected. This may be explained by the movements of birds: birds with low levels of exposure might be those staying a short time in the area and moving elsewhere (=“die”), whereas birds staying permanently in the area may have higher Pb levels. Such enhanced survival is consistent with the findings on pied flycatchers of Eeva *et al*. (2006), where males, but not females, showed a higher local survival probability in the polluted than in the unpolluted area, and of Eeva *et al*. (2009), where males, but not females, survived relatively well in heavily polluted areas. In our study, such gender-related differences in survival were not detected, but negative effects on lifetime breeding success were found only in female blackbirds.

As anticipated, we indeed showed gender-specific responses, but only for this trait. Our results thus shed light on the greater vulnerability of breeding female *Turdus merula* to Pb-induced impacts, as shown for some other bird species^35–37^. This may be due to a heavier metabolic cost and oxidative stress related to the reproductive effort, particularly the physiological interactions between Ca and Pb that notably occur during egg laying and/or the different hormonal and humoral balances under stress compared with males^32,37,54,55^. This decrease in the lifetime breeding success of female blackbirds meets the predictions of both tested hypotheses. For the most exposed females, Pb-induced stress may cause reproductive impairments (effects on offspring at any stage of breeding from egg formation and hatching to the condition and survival of the fledglings) and/or cause females to invest more in self-maintenance, with likely consequences for reproduction^35,55^. In parallel, it is also possible that this pattern mirrors changes in the males’ behaviour, with more affected individuals redirecting their energy expenditure towards their own survival and with consequences for the breeding success of their partner^32,35^. Overall, our results strongly suggest that exposure to Pb may induce a trade-off in self-maintenance versus reproduction, promoting behaviours or a reallocation of resources that could increase survival at the expense of reproductive success. Although lifetime breeding success decreased, survival increased with increasing Pb contamination. Furthermore, our analysis yielded an indication of a high cost to breeding efforts under chronic exposure to Pb, resulting in a reduced lifespan. Whereas high breeding effort appears detrimental for birds with greater Pb exposure, low breeding effort appears to be protective for the birds with lower exposure. Apart from gender-related differences in survival, our findings support the predictions of the “*stress hormone hypothesis*” and “*pollution related breeding effort*” proposed by Eeva *et al*. (2006, 2009) and Møller *et al*. (2012), in which birds are expected to show a higher survival probability but an equal or lower fledgling production in polluted areas. According to these hypotheses, pollution stress may increase stress hormone levels and/or the expenditure of antioxidants (which is in consistent with the ecotoxicological significance of the levels of Pb we measured), leading individuals to redirect their allocations towards self-maintenance and resulting in greater pollution-related impacts on females. This appears to confirm our primary hypothesis that Pb-induced stress may shape the ecology of urban birds by triggering trade-offs between life-history traits.

Life-history theory predicts fitness trade-offs, with individuals who increase their current reproductive investment suffering enhanced mortality^56^. However, such a cost of reproduction in birds is still controversial and has been shown to be sex-dependent^57^. Testing the predictions of the optimality theory of ageing on free-living jackdaws (*Corvus monedula*), Boonekamp *et al*. (2014) showed a significant cost of breeding effort regardless of gender, with actuarial senescence being greater in birds that experienced increased parental effort. The impairment of bird condition caused by chronic exposure to Pb and its effect on breeding effort could thus accelerate senescence and affect survival as found in our study. The authors also highlight that “[…] *birds can apparently cope with the increased effort for 1 year without paying an immediate survival cost*.”^58^; this could explain why we only detected a relationship for lifelong breeding success. In a recent study, it was shown that female zebra finches exposed to environmental stress exhibited both reduced breeding performance and improved survival, but the trends of such effects differed with the age of the birds^59^. The fact that environmental stress shapes the investment in reproduction and survival according to specific temporal patterns may also explain the lack of significance in our study when considering annual parameters rather than lifelong parameters.

Despite contradictory results among studies on European blackbirds, which may be due partly to the type of non-urban (farmland, woodland) and urban habitats compared and/or the measures of reproductive output considered (clutch size, brood number, fledgling production), a trend towards lower productivity but better survival in urban passerine populations appears to emerge^6–9,60^. According to Croci *et al*. (2008), it is difficult to explain why urban adapters such as the blackbird appear to have longer life expectancies than urban avoiders. They suggest that longevity may compensate for the low reproductive rates in urban birds, which appear to invest more in survival than in reproduction, and Sepp *et al*. (2017) phrased this with regards towards evolutionary processes within the pace-of-life syndrome framework.

A recent study showed that blackbirds had shorter telomeres in urban than in natural habitats, yet the rate of telomere shortening is considered an integrative measure of the ‘life stress’ experienced by an individual^61^; this argues for urban habitats as a challenge for this species. Urban avian populations have been shown to exhibit stress and different life-history and endocrine traits when compared with populations in their natural, non-urban habitats^10,62^. To explain these modifications of the ecology of urban birds, numerous factors resulting from the specific environmental conditions of urban areas have been highlighted. The impact of environmental pollution is one of these typical urban stressors and its selective role has been poorly investigated in urban ecology and physiology, but this issue is attracting more interest^15,18,22,23^.

According to the literature and to the present results, current Pb levels are low, but persistent chronic pollution may be considered a human-induced disturbance that shapes the ecology of urban wildlife. Urban environments may function as ecological traps, or even as ecotoxicological traps if pollutants such as Pb are the main drivers that render them unable to sustain sufficient productivity^63^. Although further investigations are needed to confirm the present findings and further address the underlying mechanisms and interacting responses to stressors to better understand the ecology and evolutionary trajectories in such challenging environments, the present work provides original results raising new challenges in urban ecology. This paves the way towards an integrated framework that merges ecotoxicology, ecophysiology and ecology, whose issues and methods are typically considered separately; new insights and concepts may emerge from such a comprehensive approach and cutting edge methodological perspectives.

## Materials and Methods

### Study site, blackbird survey and sampling

From 1997 to 2016, blackbirds have been surveyed in the urban park of Żeromski in Szczecin (430 000 inhabitants, NW Poland, 53°26′01N, 14°33′47E). The park (21.9 ha) is located in the city centre and is surrounded by streets and housing estates. This park was an old cemetery prior to the Second World War. Soil trace metal levels could thus be heterogeneous even over such a small area, not only because of the spatial variability of the surrounding atmospheric sources of Pb but also due to the number and composition of coffins^64^. The study area hosts a population of blackbirds with an average density of 2.5 pair/ha (for detailed description of the study area see previously published studies)^65^.

The blackbirds were surveyed throughout the entire breeding season (March 1–August 1) with one to three persons observing the behaviour of the birds for 6–8 hours (beginning at dawn) each day, so the majority nesting attempts and all fledglings were documented. Over 90% of the breeding birds were individually marked with coloured rings. The studied population is mostly sedentary (the annual return rates are 73 and 67% for males and females, respectively; 80–90% of the population is observed in the park and its vicinity during winter; and only 10–20% of the birds were never observed in or near the park between December 1 and March 1)^66^. Survival was estimated on the basis of observations after March 1. Age was determined from contrast in the wing plumage^67^. Because most of the birds were ringed as nestlings, fledglings, or in their second year of life, the exact age was known accurately for most individuals^68^. This allowed us to follow the fate of known-aged birds throughout their entire life and determine both the lifespan and the lifetime breeding success (i.e., lifetime fledgling production) for most of the sampled birds. The surveys performed on this population showed that survival probabilities (0.51 ± 0.01) and site-fidelity (0.91 ± 0.04) were high^69^, and confirmed that bird non-appearance in the subsequent breeding year is most likely related to the death of the bird. However, there are some cases in which birds disappeared from the park for more than one month during the breeding season, and there is a high probability that they could have bred successfully outside the studied park. These cases were rare and were excluded from the analyses of lifetime breeding success.

If one partner left the other partner feeding fledglings alone for at least seven days during the two weeks after the fledglings left the nest, this was considered abandonment of the last clutch. The feeding of fledglings by only one parent during a shorter period of time (less than seven days) was not considered abandonment, because parents sometimes stopped feeding fledglings after two weeks^70^. If one parent stopped feeding the fledglings after two weeks while the second parent still fed them, this was also not considered abandonment.

Feathers were sampled from 2005 to 2010 during the breeding season (from March to August) when birds were first captured for ringing, or when the birds were trapped while feeding the nestlings or fledglings (this was the case for most of the older birds). Because blackbird capture by mist-netting requires long field surveys (1-2 individuals caught/day), it was not possible to sample all the birds over a short time period. Each feather sample corresponds to one individual bird, with a total number of 115 blackbirds sampled (115 tail and 56 wing feathers). Additionally, tail feathers were sampled from blackbirds captured in 2007 in the Central Cemetery in Szczecin, a vast (172.3 ha) green urban park-like area (*n* = 20). The common blackbird begins moulting from late May to late August^71^, and the moult lasts until early September/late October, beginning approximately 15 days after the end of breeding^72^. In second-year birds, only some of the wing feathers are replaced during the first moult, but the tail feathers are completely replaced. After the second moult, no juvenile feathers remain. The level of feather contamination was similar for females and males (F_1,207_ = 1.03; *p* = 0.31, Table 1). Significant differences in contaminant levels for the two types of feathers were observed for Pb, but not for Cd, with higher Pb levels in tail than in wing feathers (F_1,207_ = 21.32; *p* < 0.0001, Table 1). To avoid any potential bias, only tail feathers were used to evaluate the relationship between life-history traits and the exposure to metals. It is thus unlikely that any age-related differences in our dataset could be biased due to the age of the feathers, variation in the moulting pattern or the contact duration of the feather with the ambient environment.

For the birds from the Żeromski Park population for which a tail feather had been collected, the following information was computed: (1) sex, (2) age in the year of feather sampling, (3) survival probability of the young birds (birds in their second year of life) in the first, second and third years after feather sampling, (4) lifespan of birds sampled in their second calendar year of life, (5) yearly breeding success and the number of broods within the year of feather sampling, (6) start and end dates of the breeding season within the year of feather sampling and abandonment of the last clutch, (7) lifetime breeding success and the lifetime number of broods.

We confirm that all methods were carried out in accordance with relevant guidelines and regulations. All experimental protocols were approved by an institutional and licensing committee as detailed hereafter. Bird capture and ringing was realized under the supervision of Dr. Dariusz Wysocki with the ringing license nr 390/201 delivered by the Polish Academy of Science. Marking (combinations of four colour rings) was performed with the permission of the Polish Academy of Sciences. At the beginning of feather sampling in 2005, no particular regulation about such sampling applied. Later, agreements for survey and feather and blood sampling were delivered by the Local Ethics Committee in Szczecin (Poland) with a renewal every three years (6/07: 26.02.2007-2009; agreement nr 12/10: 15.02.2010-2012; agreement nr: 5/13: 18.02.2013-2015). The last agreement has been delivered by the Local Ethics Committee in Poznan (Poland) since 5 April 2016.

### Analyses of trace metals

The concentrations of Cd and Pb in feathers were measured by furnace atomic absorption spectrometry (VARIAN 240Z). Prior to trace metal analyses, three alternated 5 min-soaks in acetone (pure acetone, analytical quality, Fisher Chemical) and ultra-pure water (18.2 MΩ/cm^2^) in an ultrasonic bath were used to wash the feathers to remove external contamination. None of the routinely applied feather washing methods can remove all external contamination, but an alternating deionised water and acetone bath is the one recommended methods for metals and was enhanced here with sonication^73,74^. The barbs were not removed from the feathers. Feathers were dried (45 °C) to a constant weight and weighed to the nearest 0.1 mg (dry weight, DW). Digestion was performed by dissolution in a 1:1 mixture of nitric acid (HNO_3_, 65%, analytical quality) and hydrogen peroxide (H_2_0_2_, 30%, analytical quality) in a drying oven. The samples were diluted by the addition of ultrapure water (18.2 MΩ/cm^2^). Certified reference materials (CRMs) (TORT-2 and DOLT-3, National Research Council, Canada) and blanks (acid + ultra-pure water) were prepared and analysed in the same manner as the samples. The repeatability (within-run precision) of the measurements was ensured by measuring each sample three times and checking the RSD values. Quality control and reproducibility checks were performed by regularly analysing the standard solutions used for the calibration curve, blanks and reference materials (% recovery of CRMs and standard solutions: 106 ± 12 for Cd and 102 ± 12 for Pb) between sample runs. When the measured levels of metal concentrations were below the detection limits (LoD = 0.01 and 0.27 µg.g^−1^ DW for Cd and Pb, respectively), half of the detection limit value was used for statistical analyses.

### Statistical analyses

The data obtained from the feathers sampled in the cemetery are provided to give further insight into the level of exposure to Cd and Pb in the city; they were not considered further in the statistics. All statistical analyses were performed on the blackbird population of Żeromski Park.

General linear models (GLMs) were used to identify the most important factors (year of sampling, sex and type of feather) that affected trace metal contamination. Because no significant differences in feather contamination were found between years, the data was pooled (Supplementary Information A1: Tables A1 and A2). Because Pb concentrations significantly differed between tail and wing feathers (see “Results”), the data could not be pooled, and including the factor “type of feather” was not reliable due to insufficient sample size. Thus, all further analyses were conducted on a subset of the dataset that included tail feathers only. Most of the subsequent analyses were conducted on each sex separately or included interactions between sex and explanatory factors in the models to explore potential gender-related differences.

To relate metal concentrations to age, general least square models with an added random effect were used to address the heterogeneity of variance that was detected. We used the “VarPower” variance structure (the power of the variance covariate), which was chosen from among the other structures based on the AIC criterion^75^. To investigate the potential effect of trace metal exposure on breeding success, breeding success and brood number in the year of feather sampling and lifetime breeding success or lifetime brood number were considered as dependent variables and were related to Pb and Cd concentrations including sex and interactions between these parameters. Lifetime breeding success is obviously related to the lifespan of the bird, with older birds having higher lifetime success (p < 0.001), but our purpose was to compare the lifetime breeding success (represented by total fledgling production) of different individuals with different life spans. To account for this correlation, we used Poisson regression models with total fledgling production as the dependent factor and metal concentration as the independent factor, but lifespan was included as an offset factor. To address the overdispersion that often characterizes counts, if detected, GLMs were used with a quasipoisson error distribution.

The survival analysis was performed using binomial GLMs to compute the survival probability of the young birds. The dependent variables were survival in the 1st, 2nd and 3rd year after sampling (survival coded as 1, non-survival as 0). To further investigate the effects of metals on survival, a Poisson regression between lifespan and metal concentration was performed. Overdispersion was checked and was not an issue.

We did not find benchmarks in the literature relating Pb concentrations in feathers and effects on bird health. The toxicological thresholds provided in the literature concerned blood levels such as the value of 0.2 µg/g FW for subclinical effects of Pb in birds^26^. To assess the level of Pb in feathers that may be indicative of sub-lethal effects in blackbirds (a value in feathers corresponding to 0.2 µg/g FW in blood), we quantified the relationship between Pb in blood and tail feathers using the data from studies performed on this species in cities that showed the same range of Pb levels in feathers as the present birds. For sedentary birds chronically exposed to Pb, a relatively steady relationship between Pb in blood and feathers can be expected, which was confirmed by the literature^34,38,40^. Using data from previous studies on European blackbirds^38,40^, we calculated that on average 6 µg/g Pb (DW) in tail feathers corresponded to 0.2 µg/g Pb (FW) in blood, and we considered this value as an informative threshold to compare birds exhibiting exposure levels that may or not be related to subclinical effects.

To test the hypothesis that the breeding effort of birds with the greatest exposure is related to higher costs, we compared the relationship between lifespan and Pb levels of the birds having three or more fledglings during their lifetime (considered a high breeding effort) with the birds having two or less fledglings during their lifetime (considered a low breeding effort). This threshold value of 3 fledglings was chosen based on the lifetime breeding success in the studied population: recent analyses^76,77^ show that almost half of the breeding population (47%) has a lifetime breeding success between 0 and 2 fledglings (0, 24%; 1, 14%; and 2, 9%). This test was performed separately for the most and the least contaminated birds using the threshold described above (due the small sample size, males and females were pooled).

We assumed that the longer the breeding period, the higher the investment of blackbirds in reproduction. Indeed, in the case of birds that start reproduction earlier or end later, the breeding season is longer, which allows more clutches to be produced within the breeding season. We thus consider the dates of the beginning and end of the breeding season as proxies of parental investment. We constructed normally-distributed GLMs with either first clutch or last clutch dates as dependent variables and metal concentrations as independent variables. Datasets and the residuals of the models were checked for normality and homogeneity of variance. Finally, we studied the probability of abandonment of the last clutch of the breeding season, assuming that abandonment reflects low parental investment. We related the probability of abandonment to the exposure to metals and examined whether one sex abandons the last clutch more often than the other.

Statistical analyses were run in R^78^, with the additional package “nlme”^79^. The level of significance in the statistical analyses was set at *p* < 0.05.

## Electronic supplementary material

Supplementary information

Dataset 1

## Data Availability

Data are provided as a Supplementary information file.
