# Supplementary material for: Exposure to Pb impairs breeding success and is associated with longer lifespan in urban European blackbirds
Source: Sci Rep. 2019 Jan 24;9:486. doi: 10.1038/s41598-018-36463-4 (PMC6345771; doi:10.1038/s41598-018-36463-4)
Supplement: Supplementary file 1 — Supplementary information [file 41598_2018_36463_MOESM1_ESM.pdf]

Exposure to Pb impairs breeding success and is associated with longer lifespan in urban European blackbirds

Clémentine Fritsch[1*], Łukasz Jankowiak[2] and Dariusz Wysocki[2]

[1]Chrono-environnement, UMR 6249 CNRS / University Bourgogne Franche-Comté Usc INRA, 16 route de Gray, F-25030 Besançon Cedex, France

[2]Department of Vertebrate Zoology and Anthropology, University of Szczecin, Wąska 13, 71-412 Szczecin, Poland

---

Supplementary information

A1. *Appendix 1: additional details on results*

A2. *Appendix 2: addendum to the discussion about single measurement of exposure*

Appendix 1: additional details on results

Table A1. Statistical outputs about Pb concentration in tail feathers of blackbirds and year of sampling (*n* = 115).

	Estimate	Std. Error	*t*	*p*
(Intercept)	9.334	3.278		
year2006	-3.572	3.363	-1.062	0.291
year2007	-2.681	3.315	-0.809	0.420
year2008	-3.288	3.340	-0.984	0.327
year2009	-1.588	4.636	-0.343	0.733
year2010	-2.141	3.345	-0.640	0.523

Table A2. Statistical outputs about Cd concentration in tail feathers of blackbirds and year of sampling (*n* = 115)

	Estimate	Std. Error	*t*	*p*
(Intercept)	0.175	0.152		
year2006	0.010	0.156	0.061	0.951
year2007	0.050	0.154	0.323	0.747
year2008	0.100	0.155	0.647	0.519
year2009	-0.002	0.215	-0.009	0.993
year2010	0.022	0.155	0.144	0.886

Table A3. Statistical outputs about age effect on Cd concentration in tail feathers of blackbirds (*n* = 115)

	Estimate	Std. Error	*t*	*p*
(Intercept)	0.255	0.030		
age	-0.010	0.008	-1.238	0.218

Table A4. Statistical outputs about the relationships between breeding success in the year of feather sampling and lead (Pb) or cadmium (Cd) concentrations in tail feathers with sex differences in the blackbird studied population (*n* = 108)

	Estimate	Std. Error	*t*	*p*
(Intercept)	0.248	0.607		
sex[m]	-0.069	0.090	-0.764	0.447
Pb	0.223	0.806	0.277	0.782
Cd	-0.136	1.561	-0.087	0.931
Pb:sex[m]	0.062	0.119	0.519	0.605
Cd:sex[m]	-1.249	2.772	-0.451	0.653

Table A5. Statistical outputs about the relationships between number of broods in the year of sampling and lead (Pb) or cadmium (Cd) concentrations in tail feathers with sex differences in the blackbird studied population (n = 108).

	Estimate	Std. Error	t	p
(Intercept)	0.626	0.239		
sex[m]	0.012	0.032	0.364	0.717
Pb	0.424	0.320	1.326	0.188
Cd	-0.418	0.565	-0.740	0.461
Pb:sex[m]	-0.006	0.044	-0.127	0.899
Cd:sex[m]	-0.542	1.044	-0.519	0.605

Table A6. Statistical outputs about the relationships between lifetime number of broods and lead (Pb) or cadmium (Cd) concentrations in tail feathers with sex differences in the blackbird studied population (n = 108).

	Estimate	Std. Error	t	p
(Intercept)	2.233	0.262		
sex[m]	-0.060	0.040	-1.491	0.139
Pb	-0.052	0.380	-0.136	0.892
Cd	0.434	0.648	0.670	0.504
Pb:sex[m]	0.086	0.053	1.613	0.110
Cd:sex[m]	-1.418	1.324	-1.071	0.287

Table A7. Statistical outputs about survival probability of the young birds in the 1st year after feather sampling and Cadmium (Cd) in tail feathers (n = 46)

	Estimate	Std. Error	z	p
(Intercept)	0.502	0.602		
Cd	0.532	2.193	0.243	0.808

Table A8. Statistical outputs about survival probability of the young birds in the 2nd year after feather sampling and Cadmium (Cd) in tail feathers (n = 46).

	Estimate	Std. Error	z	p
(Intercept)	-1.304	0.700		
Cd	0.098	2.510	0.039	0.969

Table A9. Statistical outputs about survival probability of the young birds in the 3rd year after feather sampling and Cadmium (Cd) in tail feathers (n = 46).

	Estimate	Std. Error	z	p
(Intercept)	-2.209	0.892		
Cd	1.257	3.011	0.417	0.676

Table A10. Statistical outputs about the relationship between Cadmium (Cd) in tail feathers and lifespan of birds sampled in their second calendar year of life (*n* = 46).

	Estimate	Std. Error	*z*	*p*
(Intercept)	1.255	0.154		
Cd	-0.011	0.554	-0.020	0.984

Table A11. Statistical outputs of the model relating lead (Pb) and cadmium (Cd) in tail feathers with the start date of the breeding season (*n* = 89).

	Estimate	Std. Error	*t*	*p*
(Intercept)	20.990	5.288		
Pb	1.134	0.691	1.641	0.104
Cd	-14.401	16.574	-0.869	0.387

Table A12. Statistical outputs of the model relating lead (Pb) and cadmium (Cd) in tail feathers with the end date of the breeding season (*n* = 89).

	Estimate	Std. Error	*t*	*p*
(Intercept)	20.990	5.288		
Pb	1.134	0.691	1.641	0.104
Cd	-14.401	16.574	-0.869	0.387

Appendix 2: addendum to the discussion about single measurement of exposure

The fact that bird exposure to metals in this study is assessed on a single measurement basis bears comment. Owing to the biology of the species (territorial and highly phylopatric species with low both breeding and natal dispersal distances [1]), and data on the studied population (mostly sedentary population, breeding and wintering areas usually stable [2–6]), most of the birds studied were reared and then bred over the same area every year, almost all individuals being resident birds living in the park throughout the year. As detailed in introduction, given (i) the temporal trends of Pb environmental emission in cities, (ii) persistent soil Pb pollution in urban areas, and (iii) Pb levels of exposure reported in *Turdus merula* from European cities within the last decade, we considered relevant to suppose that blackbirds in this work were exposed to environmental pollution levels in air, soils, and food webs that were quite steady over the study period. This is confirmed by the absence of yearly differences in Cd or Pb concentrations in our sample, which also confirms the expected chronic aspect of exposure of sedentary birds in such urban environment. According to these biological and environmental trends, the contact of blackbirds with Pb pollution in this area can be expected as chronic and constant, rendering a single measure of contamination over time appropriate for exposure assessment. Further, studies on sedentary blackbirds from reference, polluted or urban sites showed strong correlations between levels of Pb in blood and in feathers, showing that the actual individual level of exposure in a given year (measured in blood) is consistent with the level of exposure the year before (assessed in feathers) [7–9]. Beyond the additional confirmation about the chronic aspect of exposure from early life in such environments for sedentary bird populations, this suggests that although absolute levels can vary seasonally and annually, the relative differences between individuals can be maintained over time (probably because crucial factors shaping individual level of exposure such as the location of their territory and their foraging and feeding behaviour did not change). Moreover, based on significant year-to-year correlations in Cd, selenium, Pb and mercury concentrations among individuals of two species of North American sea ducks, it has been shown that single blood samples (which are reflected in single feather samples) are suitable measure of exposure for studies that address long-term effects of these metals on the birds [10]. From these points, the use of a single measure of concentrations in feathers,

which is moreover relatively integrative in time since it mirrors the exposure over all the growing period of the feather, to relate exposure to Pb and life-history traits can be thought as suitable.

Nevertheless, we acknowledge that repeated assessment of Pb (and Cd and other pollutants) exposure of individual blackbirds, between but also within years, would improve our investigations and provide a better understanding of chronic exposure patterns in cities as well as responses of the birds over short-, mid-, and long-term. This weakness might explain the lack of significance for some relationships we investigated between exposure to Pb and life-history traits, and hamper providing more straight forward insights into how and when birds trade-off between parental investment, breeding outcome and individual survival. This drawback should be addressed in further studies.

References

1. Paradis, E., Baillie, S. R., Sutherland, W. J. & Gregory, R. D. Patterns of natal and breeding dispersal in birds. *J. Anim. Ecol.* **67,** 518–536 (1998).

2. Wysocki, D. Within-season divorce rate in an urban population of Europan blackbird *Turdus merula*. *Ardea* **92,** 219–227 (2004).

3. Wysocki, D. Factors Affecting the Between-Season Divorce Rate in the Urban Populations of the European Blackbird *Turdus merula* in North-Western Poland. *Acta Ornithol.* **41,** 71–78 (2006).

4. Wysocki, D. *et al.* The size of breeding territory in an urban population of the Blackbird (*Turdus merula*) in Szczecin (NW Poland). *Ornis Fenn.* **81,** 1–12 (2004).

5. Wysocki, D. Age structure of urban population of Blackbird (*Turdus merula*) in Szczecin (NW Poland). *Zool. Pol.* **49,** 219–227 (2004).

6. Jankowiak, Ł., Wysocki, D. & Greño, J. Survival and Site Fidelity of Urban Blackbirds *Turdus merula* — Comparison of Cormack-Jolly-Seber and Barker Models. *Acta Ornithol.* **51,** 189–197 (2016).

7. Fritsch, C. *et al.* Effects of As, Cd and Pb on blackbirds: a multi-marker study. in *SETAC Europe 24th Annual Meeting, Basel, Switzerland* (2014).

8. Fritsch, C. *et al.* Influence of landscape composition and diversity on contaminant flux in terrestrial food webs: A case study of trace metal transfer to European blackbirds *Turdus merula*. *Sci. Total Environ.* **432,** 275–287 (2012).

9. Scheifler, R. *et al.* Lead concentrations in feathers and blood of common blackbirds (*Turdus merula*) and in earthworms inhabiting unpolluted and moderately polluted urban areas. *Sci. Total Environ.* **371,** 197–205 (2006).

10. Wayland, M. *et al.* Year-to-year correlations in blood metal levels among individuals of two species of North American sea ducks. *Environ. Pollut.* **150,** 329–337 (2007).
